# DNA damage and repair in Fuchs endothelial corneal dystrophy

**DOI:** 10.1007/s11033-012-2369-2

**Published:** 2012-12-29

**Authors:** Piotr Czarny, Ewelina Kasprzak, Mariusz Wielgorski, Monika Udziela, Beata Markiewicz, Janusz Blasiak, Jerzy Szaflik, Jacek P. Szaflik

**Affiliations:** 1 Department of Molecular Genetics, University of Lodz, Pomorska 141/143, 90-236 Lodz, Poland; 2 Department of Ophthalmology, Medical University of Warsaw and Samodzielny Publiczny Kliniczny Szpital Okulistyczny, Sierakowskiego 13, 03-710 Warsaw, Poland

**Keywords:** Fuchs endothelial corneal dystrophy, FECD, Oxidative stress, DNA damage, DNA repair

## Abstract

Fuchs endothelial corneal dystrophy (FECD) is a slowly progressive eye disease leading to blindness, mostly affecting people above 40 years old. The only known method of curing FECD is corneal transplantation. The disease is characterized by the presence of extracellular deposits called “cornea guttata”, apoptosis of corneal endothelial cells, dysfunction of Descement’s membrane and corneal edema. Oxidative stress is suggested to play a role in FECD pathogenesis. Reactive oxygen species produced during the stress may damage biomolecules, including DNA. In the present study we evaluated the extent of endogenous DNA damage, including oxidatively modified DNA bases, and damage induced by hydrogen peroxide as well as the kinetics of DNA repair in peripheral blood mononuclear cells of 50 patients with FECD and 43 age-matched controls without visual disturbances. To quantify DNA damage and repair we used the alkaline comet assay technique with the enzymes recognizing oxidative DNA damage, hOGG1 and EndoIII. We did not observe differences in the extent of endogenous and hydrogen peroxide-induced DNA damage between FECD patients and controls. However, we found a lower efficacy of DNA repair in FECD patients as compared with control individuals. The results obtained suggest that the lowering of the DNA repair capacity may be one of the mechanisms underlying the role of oxidative stress in the FECD pathology.

## Introduction

Fuchs endothelial corneal dystrophy (FECD, Online Mendelian Inheritance in Man [1] no. 136800) is a bilateral, often asymmetric, slowly progressive blinding disease, which was first described by Fuchs in 1910 [2]. It is estimated that FECD affects more than 4 % of the population above 40 years old and is the most common indication for corneal transplantation, the only known method of curing this disease, in many countries, including the US [3–5]. It more often affects women than men [3]. FECD can be classified as a “classic” (late-onset) or as a familiar (early-onset). The former may be subclassified as the less frequent familiar late-onset FECD and more common sporadic late-onset FECD [6]. The early-onset variant is known to be inherited as autosomal dominant disease and was found to be caused by a mutation in the *COL8A2* gene encoding the α2 subtype of collagen VIII [7, 8]. Data suggesting that the *COL8A2* mutation is responsible for late-onset FECD are not conclusive [7, 8].

FECD is characterized by the loss of corneal endothelial (CE) cells via apoptosis, abnormalities of Descement’s membrane (DM; the endothelial basement membrane) and corneal edema [10]. Although not pathognomonic, the characteristic feature of FECD is a progressive development of microscopic refractile excrescences called “cornea guttata” in the basal lamina [9, 11]. Guttea are extracellular matrix deposits secreted by the CE, which block ion transport and solute barrier functions in the endothelial layer, leading to the dysfunction of the CE [10, 12]. Guttea are the first clinical evidence of the disease, and can be detected in the fourth to fifth decade of life [13]. After this, the disease needs 10 years to develop visual symptoms. As the disease progresses, loss of CE cells and corneal edema can be observed, which eventually lead to painful epithelial and subepithelial bullae, subepithelial scarring, and profound vision loss. Because CE cells are in postmitotic rest and cannot divide in vivo, only known method to restore patients’ vision is corneal transplantation.

Despite findings about pathophysiology of FECD, little is known about the early stages of the disease. One of the proposed causes of it is oxidative stress [14, 15]. The major premise for this is fact, that CE cells are prone to oxidative stress [16]. Firstly, cornea is exposured to light since it is in the direct light path to the retina. Secondly, CE cells are in postmitotic arrest, and thirdly they constantly pump ions by Na^+^/K^+^-ATPases thus have great oxygen demand from their high metabolic activity. In patients with FECD the expression of peroxiredoxins and thioredoxin-dependent antioxidants converting hydrogen peroxide (H_2_O_2_) to water was decreased as compared to the age-matched controls [17]. Moreover, CE cells of FECD patients had an increased level of nonenzymatically glycated products, an oxidant-antioxidant imbalance, a higher accumulation of oxidative DNA damage, changes in CE cells morphology typical to oxidative stress and higher level of apoptosis as compared to control CE cells [18].

All these facts encourage us to determine whether FECD patients are more prone to oxidative DNA damage than compared to individuals without visual disturbances. To do so we measured the extent of endogenous and induced oxidative DNA damage and the kinetics of removing such damage in peripheral blood mononuclear cells (PBMCs).

## Materials and methods

### Patients

Blood samples were obtained from 50 patients with FECD and from 43 individuals with healthy corneas.

The diagnosis of FECD was based on clinical signs on the slit lamp examination (occurrence of endothelial guttae, corneal edema) and in all the cases was confirmed by the presence of characteristic lesions and polymegathism and pleomorphism of the endothelial cells on in vivo confocal microscopy (IVCM) examination [19, 20]. The control subjects had no clinical evidence of FECD and presented healthy corneal endothelium on IVCM.

All patients and controls were examined in the Department of Ophthalmology, Medical University of Warsaw (Warsaw, Poland). They underwent ophthalmic examination, including best-corrected visual acuity, intraocular pressure, slit lamp examination, fundus examination, IVCM and anterior segment optical coherence tomography including pachymetry maps (AS-OCT). The IVCM was performed by white light scanning slit confocal microscopy system (ConfoScan 3 or ConfoScan 4, Nidek Techologies, Padova, Italy). The AS-OCT was performed by Swept Source Anterior Segment Casia OCT (Tomey, Nagoya, Japan).

An informed written consent was signed by all participants and the study design was approved by the Bioethics Committee of the Medical University of Warsaw.

### Cells isolation and treatment

PBMCs were isolated from fresh samples of blood by density centrifugation (30 min, 300 × *g*) on Histopaque-1077 (Sigma-Aldrich, St. Louis, MO, USA). The number of the cells was estimated in Bürcker chamber with trypan blue to exclude dead cells. The final number of the cells in a sample was adjusted to 10^5^ cells.

PBMCs were exposed to 20 μM H_2_O_2_ for 10 min at 37 °C in phosphate buffered saline (PBS). Then, the cells were centrifuged (5 min, 300×*g*, 4 °C), resuspended in a fresh medium and left in 37 °C for 5, 10, 15, 30, 60 and 120 min of repair incubations. Cells that were not incubated (time 0) were immediately subjected to comet assay procedure. After each time of repairing incubation, PBMCs were centrifuged again and subjected to the comet assay.

### Comet assay

The comet assay was performed under alkaline version (pH > 13) according to the procedure of Singh et al. [21] with modifications [22] as described previously [23]. This version of the technique recognizes double- and single-strand breaks as well as alkali labile sites. To assess the level of oxidative DNA damage, the modified comet assay with human 8-oxoguanine DNA glycosylase (hOGG1; New England Biolabs, Ipswich, MA, USA) or Endonuclease III (EndoIII; New England Biolabs, Ipswich, MA, USA) was conducted according to the procedure described earlier [24]. hOGG1 recognizes and removes mainly 7,8-dihydro-8-oxoguanine (8-oxoguanine) when paired with cytosine, 8-oxoadenine when paired with cytosine, foramidopyrimidine (fapy)-guanine and methy-fapy-guanine [25, 26]. EndoIII recognizes and removes mainly urea, 5,6-dihydroxythymine, thymine glycol, 5-hydroxy-5-methylhydanton, uracil glycol, 6-hydroxy-5,6-dihdrothimine and methyltartronylurea [27, 28]. Aliquots of 10^5^ cells per sample were centrifuged (300×*g*, 5 min) and the supernatant was discarded. The cell pellets were gently mixed with 50 μl of 0.75 % low melting point agarose in PBS cooled to 37 °C and spread onto microscope slides precoated with 0.5 % normal melting point agarose (two gels per slide). The gels were covered with a cover slip and allowed to solidify on a cold plate for 10 min. Thereafter, the cover slips were removed and the slides immersed in a chilled lysis solution (pH 10) consisting of 2.5 mM NaOH, 100 mM EDTA, 10 mM TRIS and 1 % Triton X-100 and stored at 4 °C for 60 min. After lysis, the slides were washed three times (5 min each wash) with enzyme buffer (40 mM HEPES, 0.5 mM EDTA, 0.1 M KCl, 0.2 mg/ml bovine serum albumin; pH 8) at room temperature. A 50 μl aliquot of enzyme solution (1 U per gel) or buffer only, as control, was placed onto gel and covered with a cover slip. Enzyme-treated samples and controls were incubated in a moist chamber at 37 °C for 60 min.

Following incubation and removal of the cover slip, the slides were placed in an electrophoresis tank, DNA was allowed to unwind for 20 min in the electrophoresis buffer consisting of 300 mM NaOH and 1 mM EDTA, pH > 13. Samples that were not digested by enzymes were placed in the electrophoresis buffer immediately after lysis step. Electrophoresis was conducted in the same buffer at 4 °C for 20 min at an electric field strength of 0.73 V/cm (300 mA).

The slides were then washed in water, drained and stained with 1 μg/ml DAPI and covered with cover slips. The slides were examined at 200 × magnification in an Eclipse fluorescence microscope (Nikon, Tokyo, Japan) attached to COHU 4910 video camera (Cohu, San Diego, CA, USA) equipped with a UV-1 filter block consisting of an excitation filter (359 nm) and a barrier filter (461 nm) and connected to a personal computer-based image analysis system, Lucia-Comet v. 5.41 (Laboratory Imaging, Praha, Czech Republic).

Fifty images were randomly selected from each sample and the percentage of DNA in the tail of comets (% tail DNA) was measured. The mean value of the % tail DNA in a particular sample was taken as an index of the DNA damage in this sample. All experiments were performed in duplicate. The results obtained for hOGG1 and EndoIII were normalized by subtracting the level of DNA damage for the buffer alone.

### Statistical analysis

All the values in this study were expressed as mean ± SEM from two separate experiments. If group had normal distribution, as assessed by Shapiro–Wilk test, the differences between means were evaluated by applying Student’s *t* test, otherwise the Mann–Whitney test was used. The data were analyzed using the STATISTICA (StatSoft, Tulsa, OK) statistical package.

## Results

### Basal endogenous DMA damage

**Fig. 1:**
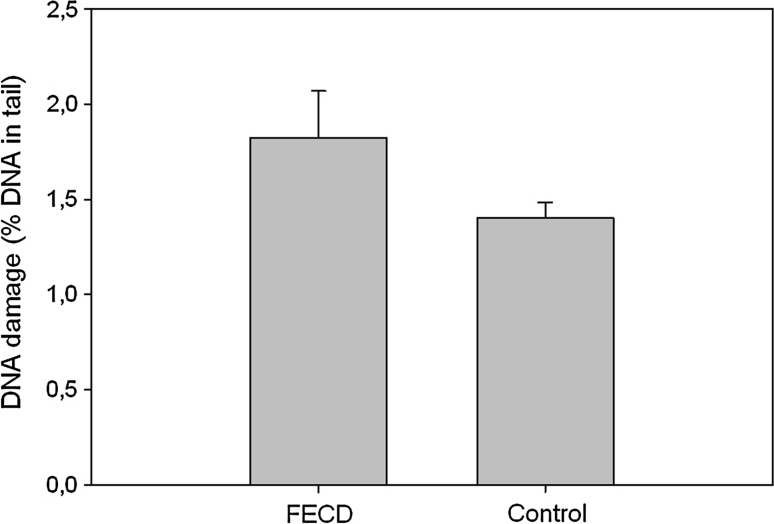
Mean basal endogenous DNA damage by the alkaline comet assay as the mean of percent DNA in comets’ tails of peripheral blood mononuclear cells of patients with Fuchs endothelial corneal dystrophy (FECD) and individuals without visual disturbances (control). 50 FECD patients and 43 controls were analyzed. The number of cells scored for each individual was 100 and the analysis was repeated two times. *Error bars* denote SEM

Figure 1 presents mean basal endogenous DNA damage in PBMCs of patients with FECD and controls without visual disturbances measured by alkaline comet assay as a percent of the DNA in the tail. Although we observed a higher extent of DNA damage in patients with FECD than in the controls, there was no statistical significance between these two groups.

### DNA damage induced by hydrogen peroxide

**Fig. 2:**
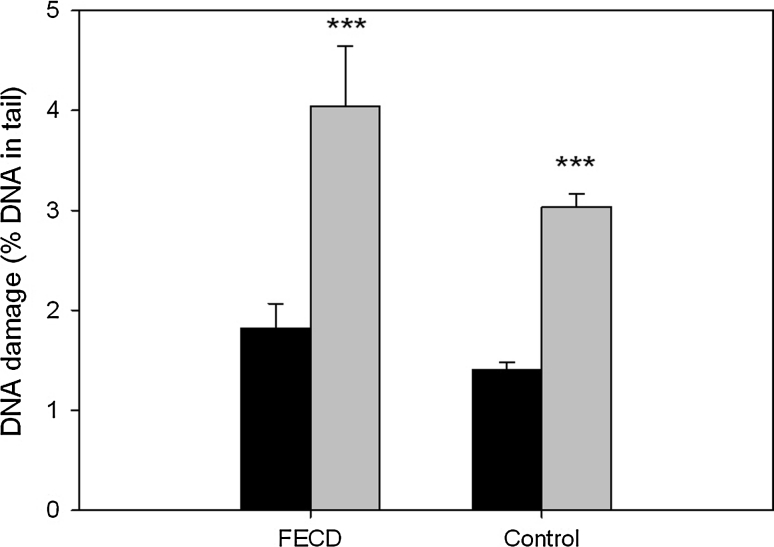
Mean basal endogenous DNA damage (*black bars*) and mean DNA damage evoked by H_2_O_2_ (*grey bars*) measured by the alkaline comet assay as the mean of percent DNA in comets’ tails of peripheral blood mononuclear cells of patients with Fuchs endothelial corneal dystrophy (FECD) and individuals without visual disturbances (control). 50 FECD patients and 43 controls were analyzed. The number of cells scored for each individual was 100 and the analysis was repeated two times. *Error bars* denote SEM, ***p < 0.001, as compared with the untreated cells

Figure 2 displays mean DNA damage induced by 10 min incubation with H_2_O_2_ in PBMCs of patients with FECD and control group without this disease measured by alkaline comet assay. For both groups there was a significant increase of DNA damage as compared to the untreated cells (*p* < 0.001). To measure the susceptibility of DNA of PBMCs to H_2_O_2_ we calculated the relative increase in the DNA tail. This increase was equal 221.8 % for the patients with FECD and 216.3 % for the control. The difference in the DNA susceptibility to hydrogen peroxide in patients and controls PBMCs was not great enough to be statistically significant.

### Oxidative modifications to the DNA bases

**Fig. 3:**
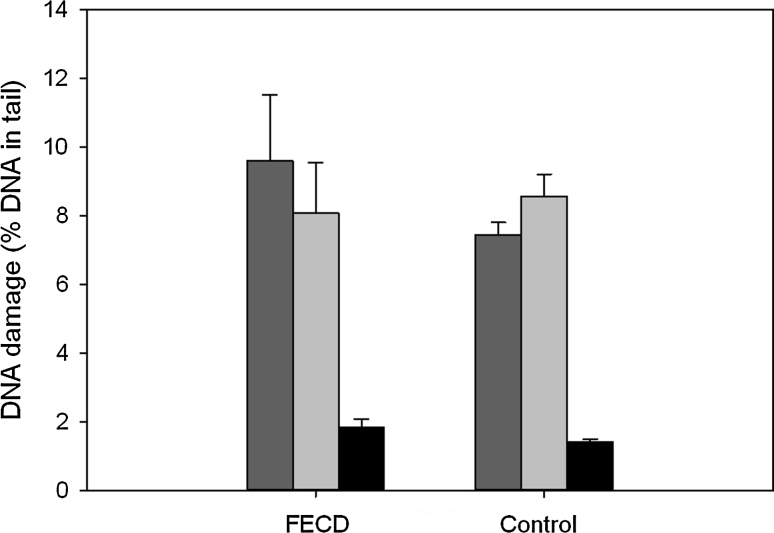
Mean basal endogenous (*black bars*) and endogenous oxidative DNA damage recognized by endonuclease (*light grey bars*) and human 8-oxoguanine glycosylase 1 (*dark grey bars*) measured as the mean percentage of DNA in comet tails of peripheral blood mononuclear cells of patients with Fuchs endothelial corneal dystrophy (FECD) and individuals without visual disturbances (control). The results obtained for the enzymes were normalized by subtracting the level of DNA damage observed for the enzyme buffer only. The number of cells scored for each individual was 100 and the analysis was repeated two times. *Error bars* denote SEM

Figure 3 displays mean percent DNA in the tail in PBMCs of FECD patients and controls measured by modified version of alkaline comet assay with hOGG1 or EndoIII, reduced by mean percent DNA in the tail evoked by the enzyme buffer only. This allowed us to analyze these modifications of the DNA bases, which are recognized by specific enzymes, but are not recognized in non-modified alkaline comet assay. Results do not show statistically significant difference in percent DNA in tail between FECD patients and controls.

### DNA repair

**Fig. 4:**
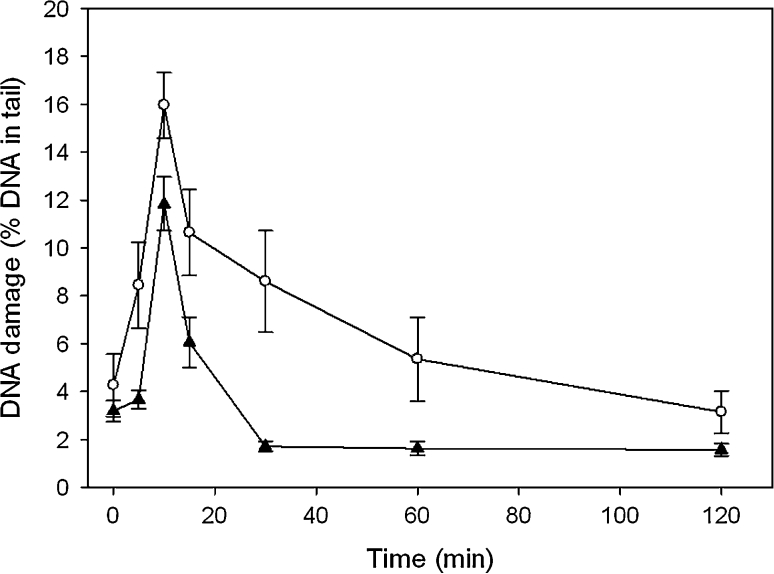
Representative kinetics of DNA damage repair in peripheral blood mononuclear cells of one chosen patient form 50 individuals with Fuchs endothelial corneal dystrophy (*open circle*) and one individual selected from 43 controls without vision disturbances (*filled up-right triangle*). DNA damage was induced by incubation with 20 μM hydrogen peroxide for 10 min at 37 °C and was measured by alkaline comet assay. Repair incubation was in a fresh medium after washing out hydrogen peroxide. *Error bars* denote SEM

**Fig. 5:**
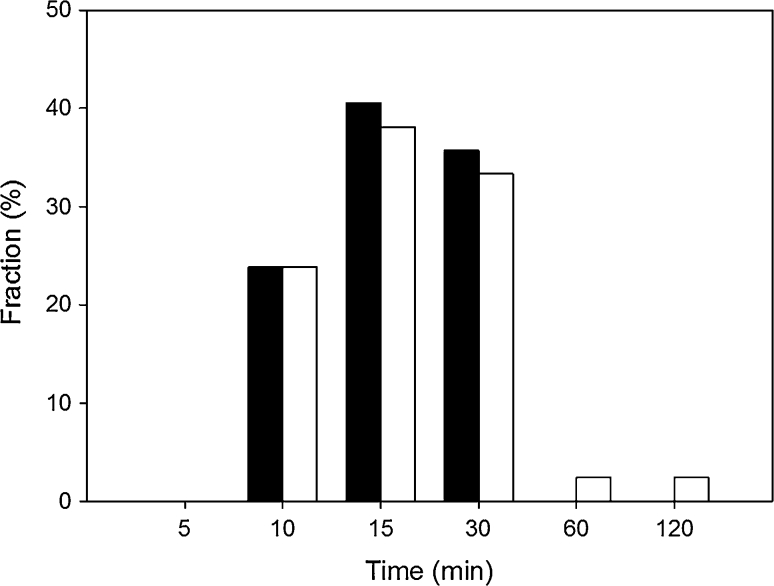
Distribution of time during repair incubation, at which the maximal DNA damage was observed in patients with Fuchs endothelial corneal dystrophy (*black bars*) or individuals without vision disturbances (*white bars*). DNA damage was induced by incubation with 20 μM hydrogen peroxide for 10 min at 37 °C and was measured by alkaline comet assay. Repair incubation was in a fresh medium after washing out hydrogen peroxide. The number of cells scored for each individual was 100 and the analysis was repeated two times

We assessed DNA repair in individuals with FECD and controls by exposure of their PBMCs to 20 μM hydrogen peroxide and evaluation of DNA damage immediately after the exposure and after 5, 10, 15, 30, 60 and 120 min of repair incubation. Figure 4 displays such kinetics of DNA damage repair. Hydrogen peroxide evokes mainly DNA single-strand breaks and oxidative modifications of the bases [29]. Most of the latter ones are not recognized by alkaline comet assay and are repaired mainly by nucleotide excision repair (NER) and base excision repair (BER). In these two repair pathways initial step includes introduction of DNA breaks or excision of the modified base, thus creating structures which can be recognized by alkaline comet assay. This is why, when we monitor the kinetics of the DNA repair, we can observe initial rapid elevation DNA damage as a consequence of DNA repair enzyme activity. Figure 5 presents time of repair incubation, at which maximal DNA damage occurred. Both the patients and individuals without vision disturbances had most often maximal DNA damage after 15 min incubation.

**Fig. 6:**
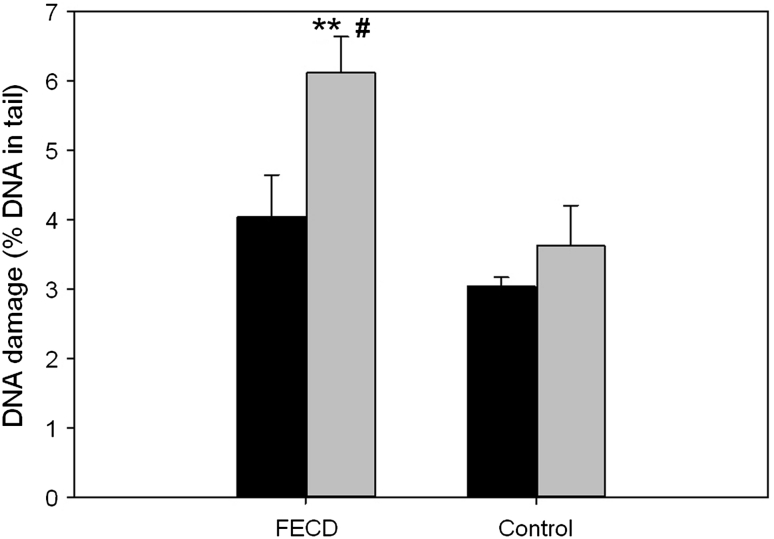
Efficiency of the DNA damage repair in peripheral blood mononuclear cells of individuals without vision disturbances (control) and patients with Fuchs endothelial corneal dystrophy (FECD). DNA damage induced by 20 μM hydrogen peroxide for 10 min in 37 °C was measured by the alkaline version of comet assay as the mean of percent DNA in the comets tails after washing out hydrogen peroxide at 0 (*black bars*) and 60 min (*grey bars*). *Error bars* denote SEM; **p < 0.01, as compared with the control; ^#^ *p* < 0.05, as compare to the time 0 min

Figure 6 shows mean percent of DNA in the tail of PBMCs of patients with FECD and controls measured by alkaline comet assay after incubation with hydrogen peroxide (time 0) and after 60 min repair incubation. Since after cessation of the exposure all subsequent procedures were made at 4 °C and the cells were washed with cold buffer the extend of DNA damage at time 0 reflects hydrogen peroxide susceptibility of the cells. We did not find any statistically significant difference between patients and controls at this time. On the other hand, DNA damage in PBMCs of patients with FECD after 60 min incubation was significantly higher than compared to the DNA damage in the patient cells at 0 time, in contrast to the control cells, where at both times DNA damage was at the same level. Moreover, DNA damage in the patients PBMCs after 60 min of the repair incubation was significantly higher when compared to the DNA damage in the controls cells at the same time. These results indicate a lower efficacy of oxidative DNA damage repair in FECD patients than in the controls.

## Discussion

The aim of our work was to evaluate the extent of endogenous DNA damage, the susceptibility to oxidative DNA damage induced by hydrogen peroxide and efficiency of DNA damage repair in patients with FECD as compared to the control group without vision impairment. Although our research should have been done on corneal cells since importance of cornea in development of the disease, we have chosen PBMCs. There are several reasons for that. Firstly, corneal cells are not so easily accessible as PBMCs. Besides that, PBMCs are affected by environmental condition, which causes oxidative DNA damage in the cornea. Moreover, research done on PBMCs can reveal evidence of inherited defect in DNA damage and repair, because their genetic constitution reflects the constitution of whole organism.

We found that basal endogenous DNA damage in patients with FECD was not statistically significant higher than compared to controls. We also did not observe difference in susceptibility to hydrogen peroxide-induced DNA damage of PBMCs from the patients than of PBMCs isolated from the controls. On the other hand, there was a significantly higher DNA damage extent in the patient PBMCs 60 min after exposure to hydrogen peroxide when compared to the control PBMCs. This could indicate the impairments of oxidative DNA damage repair pathways in patients with FECD. On the other hand, we did not observe elevated levels of oxidative DNA damage in individuals with the disease. This may be due to low level of oxidative stress in these cells, at which repair efficiency in both patients and controls is sufficient enough not to allow accumulation of the oxidative DNA damage to occur. Furthermore, due to high oxidative stress in cornea, even small impairments of DNA repair systems may have multiplied effects, leading to accumulation of DNA damage and development of the disease.

As mentioned earlier, hydrogen peroxide induces DNA breaks and oxidative modifications of the DNA bases. Latter ones are repaired mainly through BER and NER. In these two repair pathways we can distinguish four steps: recognition of the DNA damage, excision, DNA synthesis and ligation. This increase of DNA damage during the repair incubation due to the actions of excision enzymes introducing DNA breaks or apurinic/apyrimidinic site (AP site) recognized by alkaline comet assay, was mostly observed between 10 and 30 min after the exposure for both patients and controls (Fig. 5). Furthermore, most of the patients and controls had maximal DNA damage after 15 min of the repair incubation. This may indicate that the efficiency of the recognition and excision steps in the patients and controls were at a similar level. Therefore, we could assume that the observed differences between kinetics of FECD patients and controls are results of impairment of the synthesis and ligation steps of BER or NER. On the other hand, our not yet published results from genotyping of the *hOGG1* showed a higher occurrence of the Ser326Cys polymorphism in FECD patients than in the controls.

Since studied group was relatively small and was ethnically homologous, our results, although statistically significant, should be considered as preliminary and there is a need for more complex research including molecular, clinical, and epidemiologic aspects.

In conclusion, we hypothesize that lower efficiency of oxidative DNA damage repair pathways in the patients comparing to the controls, may contribute to development of FECD, especially when oxidative stress is one of the postulated mechanism of pathogenesis of the disease.
